# An Ultrawideband Polarization-Insensitive Diffusion Metasurface Using Period Changed Unit Cell for RCS Reduction

**DOI:** 10.3390/ma14175053

**Published:** 2021-09-03

**Authors:** Jianzhong Chen, Chengwei Zhang, Yutong Zhao, Lei Lin, Liang Li, Tao Su, Bian Wu, Jinshan Ding

**Affiliations:** 1 National Key Laboratory of Antennas and Microwave Technology, Xidian University, Xi’an 710071, China; jianzhong.chen@xidian.edu.cn (J.C.); zhangchengwei1996@126.com (C.Z.); flymanllm@aliyun.com (L.L.); taosu@mail.xidian.edu.cn (T.S.); bwu@mail.xidian.edu.cn (B.W.); ding@xidian.edu.cn (J.D.); 2 Nanjing Research Institute of Electronics Technology, Nanjing 210039, China; linleimail@126.com

**Keywords:** RCS reduction, polarization insensitive, ultrawideband, diffusion metasurface

## Abstract

A polarization-insensitive diffusion metasurface using a period-changed unit cell is presented for reducing the radar cross-section (RCS) of metallic objects in ultrawideband. Two metallic Minkowski loops are proposed as coding elements, different from traditional designs. The “0” element is constructed by period-changed unit cells to achieve a 180 ± 30° phase difference with the same reflection amplitude of nearly −0.9 dB in an ultrawideband from 7.1 to 29.2 GHz. Multilayer geometry with a thickness of 4.5 mm (about 0.105λ_0_ at the lowest operating frequency) and rotational symmetry loops are used to realize the ultrawideband characteristic and polarization-insensitive behavior. For verification, a polarization-insensitive diffusion metasurface is designed, fabricated, and measured. The simulated and measured results of the diffusion metasurface are in good consistency and the results both show that the metasurface enables a 10 dB backscattering reduction over an amazing ultrawideband ranging from 7.1 to 29.2 GHz (BW of 122%).

## 1. Introduction

With the rapid development of radar detectors, the requirements for electromagnetic (EM) stealth are becoming more and more important and challenging. The key to these technologies is to achieve backscattering reduction [1,2,3,4,5,6,7,8,9,10,11,12,13,14,15,16,17,18,19]. There are two technical methods to reduce the RCS, which is defined as the equivalent area of a target as seen by the radar. One of them is an absorptive method that converts scattered energy into heat [3,4,5], and the temperature change causes the target to be detected by infrared detectors. The other approach is to use the coding metasurface to diffuse the reflected energy in other directions [6,7,8,9,10,11,12,13,14,15,16,17,18,19]. The coding elements of the metasurface are designed with different phases and the same reflection amplitude for normal incidence. After the optimization [5] of an annealing algorithm, the backscattered wave of the coding metasurface with a specific sequence is diffused to other directions through destructive interference, resulting in a lower backscattered RCS.

Because of its powerful electromagnetic (EM) wave-processing capability, a metasurface composed of subwavelength structures has been widely used in recent years. According to the generalized Snell’s law, the propagation direction of electromagnetic waves can be controlled by introducing a sudden phase change at the interface.

The applications of metasurfaces are diverse. In addition to electromagnetic stealth, there are other applications for metasurfaces such as the realization of a low-profile bandpass frequency-selective surface [6], a low-cost nonuniform metallic lattice for rectifying the aperture near-field of electromagnetic bandgap resonator antennas [7], directivity improvement of a Fabry–Perot cavity antenna by enhancing near-field characteristics [8], and gain enhancement of wideband circularly polarized UWB antenna using FSS [9]. Recently, the possibility of the realization of strong plexciton dynamics and pronounced vacuum Rabi oscillations in toroidal plasmonic metasurfaces was presented by Arash et al. [10]. In addition, metasurfaces have successfully been used for reconfiguring radiation patterns of different antennas. A captive single-layer frequency selective surface (FSS) was proposed by Das et al. to realize the pattern reconfigurability of a monopole antenna producing an end-fire-like beam pattern in which the working principle of the FSS is theoretically supported by pattern multiplication analysis [11].

It is worth noting that metasurfaces can be made of all-dielectric all-metal materials and printed surfaces. Metasurfaces can be made of all-dielectric materials as explained in additively manufactured perforated superstrate to improve directive radiation characteristics of the electromagnetic source, and a high-gain wideband EBG resonator antenna for a 60 GHz unlicensed frequency band [12]. In addition, metasurfaces can be made of all-metal structures as explained in all-metal wideband metasurfaces for the near-field transformation of medium- to high-gain electromagnetic sources [13].

To reduce the backscattering RCS, a checkerboard metasurface composed of a perfect electric conductor and artificial magnetic conductor is proposed. However, the metasurface usually behaves as a limited band because the reflection phase of the PEC is constant.

The other method is to use a metasurface composed of two AMC structures working at different frequencies to obtain a wider working frequency bandwidth.

Achieving broadband RCS reduction and controlling the characteristics of dispersed EM fields have always been a big challenge. The research to obtain broadband [14,15,16,17] and efficient RCS reduction performance is one of the hotspots of current investigation and also the focus of future research.

In this paper, a polarization-insensitive diffusion metasurface constructed with different period elements is proposed for ultrawideband RCS reduction. For different polarizations, the metasurface resulted in a significant RCS reduction in the frequency range from 7.1 to 29.2 GHz (approximately 122% BW) at normal incidence. Moreover, bistatic RCS reduction was achieved using the proposed metasurface. Finally, a 1-bit coding metasurface composed of 12 × 12 super cells was designed, fabricated, and measured. Both simulation and measured results show that the polarization-insensitive diffusion coding metasurface effectively manifested an ultrawideband and multiple-beam diffusion.

## 2. Design Principle of Coding Metasurface

The 1-bit coding metasurface with the function of backscattering reduction is designed to turn the specularly reflected energy into diffusion reflection of multiple beams. The scattering field of the coding metasurface can be calculated by antenna array theory [18,19].

In an array, the array factor (AF) is a composite signal formed by the coherent superposition of the signals on the array after being received by each array element. It can be expressed as Equation (1):

(1) $$\begin{array}{l} \mathrm{AF}=\sum_{m=1}^{M}\sum_{n=1}^{N}A_{\left(m,n\right)}\exp\left\{j\left[k_{0}P_{x}\sin\theta \cos\varphi +\right.\right.\left.\left.k_{0}P_{y}\sin\theta \sin\varphi +\psi_{m,n}\right]\right\} \end{array}$$

The array factor cannot describe the spatial response of the array, and the pattern of each element (EP) describes the spatial response of the element. When modeling the element pattern, it is often expressed in the exponential form of the cosine function, and its exponential form is called the element factor. The formula of the element pattern EP is shown as Equation (2):

(2) $$\mathrm{EP}=\cos^{\frac{EF}{2}}\theta$$

The synthetic pattern expression of the entire array can be obtained by pattern multiplication—that is, the product of the array element pattern EP and the array factor AF.

Assuming that the coding metasurface is formed by *M* × *N* coding elements, each array element is a subarray, which is only formed by the “0” element or the “1” elements, and the distribution of “0” and “1” basic elements is arbitrary. Assuming that the reflection phase of the (*m*, *n*) element is *φ*(*m*, *n*), according to the far-field approximation condition, when the incident plane wave illuminates the coding metasurface vertically, the far-field scattering over the metasurface can be expressed as Equation (3):

(3) $$\begin{array}{l} F(\theta ,\varphi )=EP\cdot AF\\ =\cos^{\frac{EF}{2}}\theta \cdot \sum_{m=1}^{M}\sum_{n=1}^{N}A_{\left(m,n\right)}\exp\left\{j\left[k_{0}P_{x}\sin\theta \cos\varphi \right.\right.\left.\left.+k_{0}P_{y}\sin\theta \sin\varphi +\psi_{m,n}\right]\right\} \end{array}$$

where *θ* and *φ* are respectively the elevation and azimuth direction of scattering. When the elevation angle *θ* is equal to 0° or 180°, Equation (1) can be simplified as

(4) $$F(0^{\circ }\mathrm{or}\;180^{\circ },\varphi ){=I}_{0}\sum_{m=1}^{M}\sum_{n=1}^{N}A_{\left(m,n\right)}e^{j\psi_{m,n}}$$

For the metal plate with the same dimensions over the metasurface, the reflection magnitude *A* = 1 and the reflection phase is a constant value of 180°. The scattering field of a metal plate of equal size in the incident direction can be described as

(5) $$F_{metal}=\left|M\times N\right|$$

The following calculation functions for RCS backward scattering are based on antenna theory [20]. RCS reduction concerning the same dimensions of the metal plate can be expressed as

(6) $$\mathrm{RCS}\;\mathrm{reduction}=20\log_{10}\left|\frac{\sum_{m=1}^{M}\sum_{n=1}^{N}A_{\left(m,n\right)}e^{j\psi_{m,n}}}{M\times N}\right|$$

Because the encoding unit amplitude of the metasurface is a total reflection and the number of the two encoding units is equal, the RCS reduction can be simplified as

(7) $$\mathrm{RCS}\;\mathrm{reduction}=20\log_{10}\left|\frac{1+e^{j\left(\varphi_{1}-\varphi_{2}\right)}}{2}\right|$$

To achieve a 10 dB RCS reduction, the phase difference condition is illustrated through specific transformation steps as follows (Equation (8)):

(8) $$\begin{array}{l} 2+2\cos\left(\varphi_{1}-\varphi_{2}\right)\leq 0.4\\ \;\;\;\;\varphi_{1}-\varphi_{2}\leq 180^{{}^{\circ}}\;\pm \;37^{{}^{\circ}} \end{array}$$

RCS reduction calculated by antenna array theory based on a scattering field in far-field requires at least two types of elements satisfying the reflection phase difference criteria of 180° ± 37°, where *φ*_1_ and *φ*_2_ are reflection phase coefficients of coding elements type “0” and “1,” respectively. By changing the coding sequence of the metasurface, the incident electromagnetic energy is scattered in all directions, forming as many beams as possible.

## 3. Results and Discussion

### 3.1. Element Design

**Figure 1:**
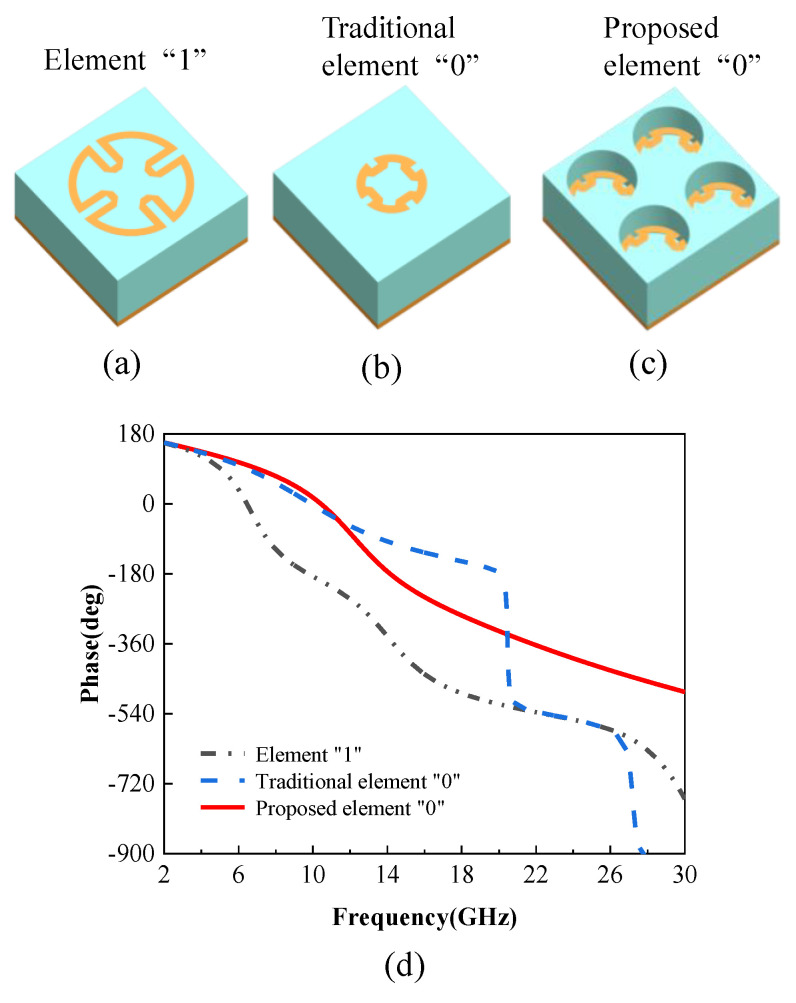
The traditional coding element of the diffusion metasurface. (**a**) The three-dimensional (3D) view of the “1” element. (**b**) The 3D view of the traditional “0” element. (**c**) The 3D view of the proposed period-changed “0” element. (**d**) Phase contrast between the “1” element and traditional “0” element, and the proposed period-changed “0” element.

The traditional coding metasurfaces are usually constructed with two basic coding elements with the same period that can generate opposite phases, and the bandwidth is limited. Figure 1a,b show the three-dimensional (3D) view of the “1” element, the traditional “0” element, and the proposed “0” element, respectively. The elements consist of three parts: a dielectric layer, a metallic Minkowski loop structure, and a metallic background, a scheme that confirms a complete reflection. The Minkowski rings are rotationally symmetric, which is the key to polarization-insensitive EM scattering. The selected dielectric substrate is F4B [21] with a dielectric constant of 2.2, thickness of 4.5 mm, and loss tangent of 0.002. The phase properties of the two traditional coding elements are shown in Figure 1d. It can be seen that the phase of the traditional element “0” drops sharply at 20.7 GHz.

Different from traditional metasurface element structure design [5,14,15,16,18,22,23,24,25], we propose a 180° phase difference between the two elements in a wider frequency band, which is the attribute to the period-changed unit cell shown in Figure 1c. An idea is proposed to make the cell size, period, and dielectric thickness of the “0” element become one-half of the dimensions inside the “1” element, and 2 × 2 “0” unit cells are treated as a “0” element. The advantage of this method is the introduction of dual-resonance mode. The resonance frequency of the “0” element will be shifted to the higher frequency (the smaller the dimension, the higher the resonance frequency). In this way, the phase of the period-changed “0” element changes slowly at 20.7 GHz, and the bandwidth of the 180° phase difference between the “1” element and the period-changed “0” element could be expanded as quadruple bandwidth, as shown as in Figure 1d.

**Figure 2:**
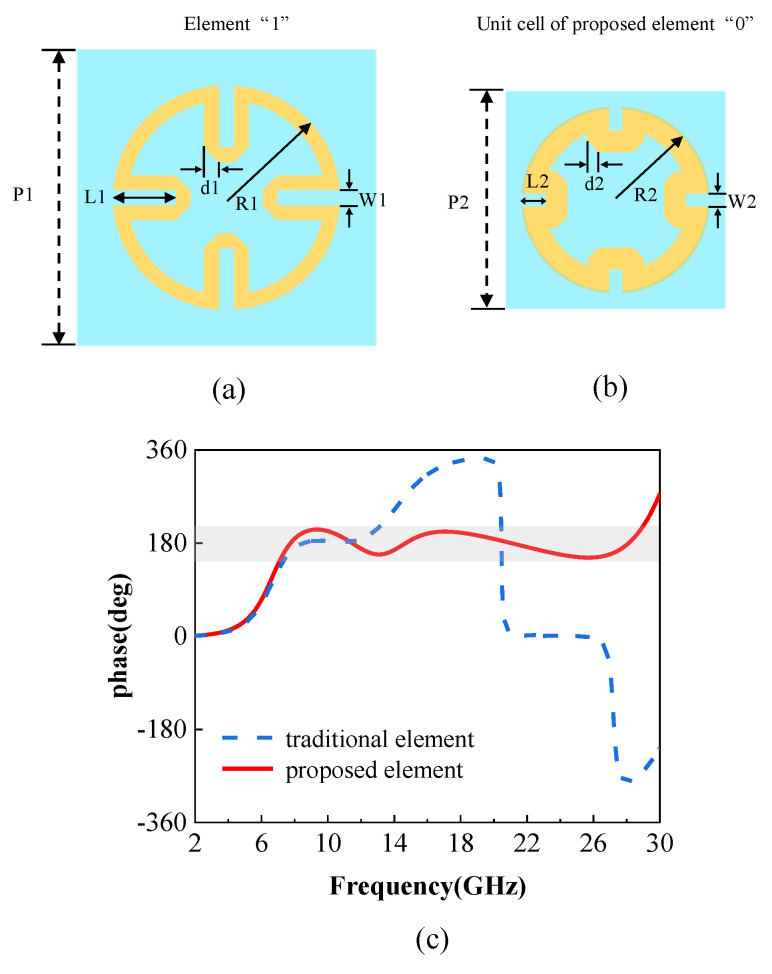
The proposed period-changed coding element and phase difference. (**a**) Dimensions of the “1” element, (**b**) dimensions of the “0” unit cell of the proposed period-changed “0” element (dimensions: P1 = 10 mm, P2 = 5 mm, w1 = 0.5 mm, w2 = 0.3 mm, d1 = 0.5 mm, d2 = 0.25 mm, R1 = 3.8 mm, R2 = 2.15 mm, l1 = 2.09 mm, l2 = 0.54 mm), and (**c**) phase difference of traditional coding elements and proposed period-changed coding elements.

To obtain the desired reflection phase and amplitude properties, the geometric parameters of the unit cell are swept by ANSYS Electronics Desktop 19.0. It is worth mentioning that those coding elements should be set to master–slave boundaries and be excited by the Floquet port, which is important for obtaining the phase properties in periodic conditions. The geometric parameters are finally chosen as depicted in Figure 2a,b. Figure 2c shows the simulated phase difference of traditional coding elements and proposed period-changed coding elements. The phase-difference bandwidth of traditional coding elements is nearly from 6 GHz to 13 GHz within 180° ± 30°, whereas the phase difference of proposed period-changed coding elements is approximately 180° in an ultrawideband. In particular, from 6 GHz to 28 GHz, the phase difference ranges from 150° to 210°. The bandwidth of the proposed element is twice that of the traditional element, which proves the proposed idea.

### 3.2. Coding Matrix Arrangement

The surface of a traditional conductor has a uniform reflection phase when a plane wave is incident, resulting in strong directional scattering. To control the direction of the reflected beam, a phase gradient is introduced in the interface. Here, our goal is to redirect the scattering energy in all directions to minimize directional reflection. The reflection phase of each part of the surface should be distorted as much as possible, rather than equidistant or with a gradient shift. The simplest way is to generate a matrix of random phase distribution. We generated a phase matrix containing 0 and 1 composed of opposite reflection phases (0° or 180°).

**Figure 3:**
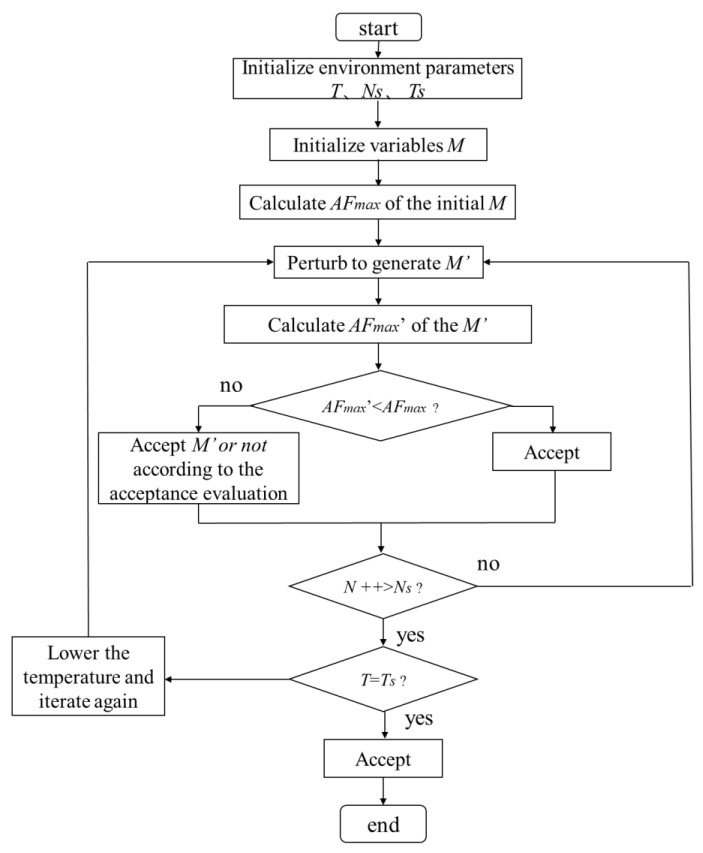
Flow chart of the annealing algorithm to find the optimal coding matrix (*M_best_*) that leads to the desired scattering field with the smallest (*AF_max_*) value.

Here, an annealing algorithm was employed to find the optimal coding matrix due to its high efficiency. A flow chart of the annealing algorithm is shown in Figure 3. The main parameters of the annealing algorithm are the initial temperature T, the final temperature Ts, the number of iterations I, the total number of iterations Is, and coding matrix M.

**Figure 4:**
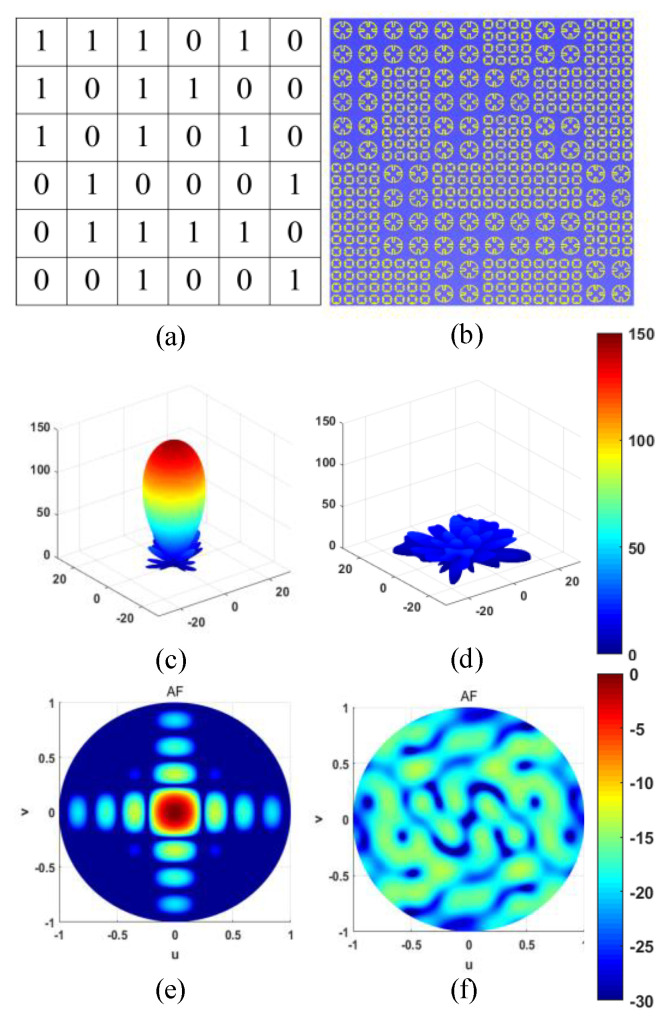
Matrix arrangement and calculated scattering fields. (**a**) Schematic diagram of super cells. (**b**) The sequence of the coding metasurface sample, with scattering fields calculated at the center frequency of 18 GHz. (**c**,**d**) Scattering 3D patterns of the uniform coding matrix and the optimal coding matrix, respectively. (**e**,**f**) Scattering fields on the wave vector domain of the above two matrixes, respectively. (AF: a composite signal formed by coherent superposition of the signals on the array after being received by each array element).

Here, one kind of coding metasurface is presented with a particular sequence that is optimized by the simulated annealing algorithm to be optimal for RCS reduction. To improve the performance of the unit structure, a concept of super cell coding was proposed. A super cell “1” contains 2 × 2 element “1,” and a super cell “0” contains 2 × 2 element “0.” Figure 4a shows the schematic diagram of super cells, where the metasurface is arranged by binary super cells “0” and “1.” The sequence of the coding metasurface sample is shown in Figure 4b, which covers an area of 240 mm × 240 mm. In fact, each super cell contains four “1” elements, and super cell “0” contains 16 basic “0” unit cells. According to the antenna array pattern theory [18,19], we took the scheme of the coding sequence as the antenna array factor to synthesize the diffuse reflection pattern. To make the reflection pattern as multi-beam as possible and the amplitude value as low as possible, we used the annealing algorithm to optimize the coding matrix (phase of the array factor) to get the ideal radiation pattern form. Similarly, a reflection characteristic map with a metal plate of the same size as the code array was also synthesized. As shown in Figure 4c–f, a comparison between the diffuse reflection characteristics of the coding sequence and the total reflection characteristics of the metal backplane can be seen. The presented sequence featured excellent scattering characteristics.

**Figure 5:**
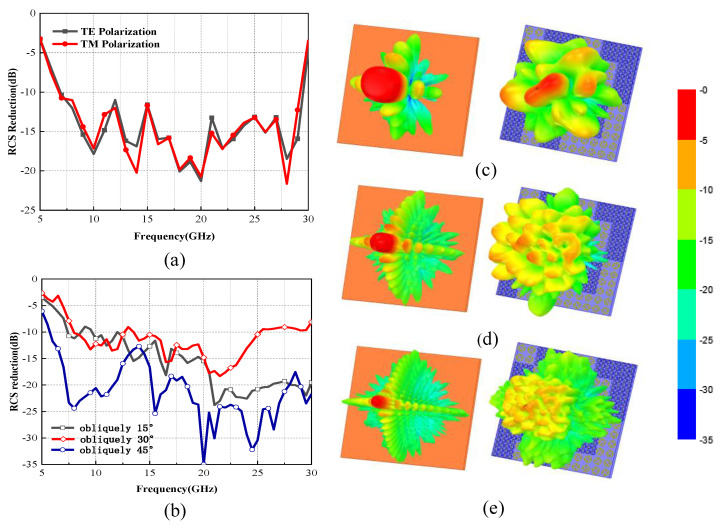
(**a**) Simulated RCS reduction of TE and TM polarizations under normal incidence. (**b**) Bistatic RCS reduction of different incident angles. 3D scattering patterns of the metal plates and the diffusion metasurface under the normal incidence of TE polarization at (**c**) 8 GHZ, (**d**) 18 GHz, and (**e**) 29 GHz.

A metasurface with a specific arrangement was also designed and simulated in ANSYS Electronics Desktop 19.0. A diffusion metasurface should be conducted with a PML (perfectly match layer) boundary condition to ensure that there is no effect of reflected waves on the metasurface in a smaller space compared to the radiation boundary conditions. In addition, the coding elements or diffusion metasurface should be set to either a broadband or multi-frequency analysis setup solution for accuracy of the ultrawideband property. The simulated results of the monostatic radar cross-section of the metasurface under a normal incident of TE and TM polarizations across a frequency of 7 GHz to 30 GHz is shown in Figure 5a. The TE- and TM-polarized RCS reduction results are in good consistency with each other, indicating that the metasurface showed ultrawideband and polarization-insensitive properties. The slight difference between TE and TM polarizations in the results was considered to be caused by the step size of the frequency sweep in the simulation process.

The 3D scattering patterns of the metal plates and the diffusion metasurface under the normal incidence of TE polarization at 8 GHZ, 18 GHz, and 29 GHz were studied and are illustrated in Figure 5c–e. Compared with the reflection results of same-size metal plates, the diffusion behaviors of the proposed coding metasurface can be clearly observed. The incident EM waves were scattered in all directions when applying the proposed metasurface, diffusing into multiple beams.

The bistatic RCS reduction properties of the metasurface were also investigated with the plane waves obliquely incident from 15° to 45°. As we can see in Figure 5b, the bistatic RCS reduction of the metasurface was higher than 10 dB from 7 to 28 GHz, showing an excellent broadband property. As the incident angle increased, the bandwidth decreased slightly due to the unconformity of the relationship between the phase shift and patch length when the wave was obliquely incident. However, low RCS properties were still observed in an ultrawideband for the proposed metasurface.

**Figure 6:**
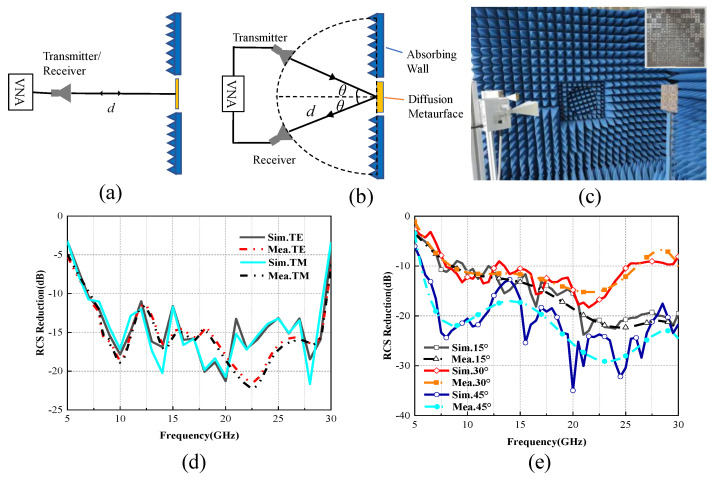
Prototype of the diffusion metasurface, measurement setup, and measured results. (**a**) Monostatic and (**b**) bistatic measurement setup, and (**c**) test environment and measurement setup. (**d**) Simulated and measured monostatic RCS reduction of the proposed diffusion metasurface at normal incidence under TE and TM polarizations. (**e**) Simulated and measured bistatic RCS reduction of the proposed diffusion metasurface at oblique incidence (15°, 30, 45°) under TE polarization.

For the monostatic RCS measurement, the sample was measured in an anechoic chamber, where a horn antenna as transmitter and receiver was placed perpendicularly to the sample to ensure vertical incidence, and the top view of measurement settings are displayed in Figure 6a. Likewise, the top view of the bistatic RCS measurement setup is shown in Figure 6b, where the two horns were respectively connected to two ports of the vector network analyzer (Anritsu MS46322A). The direction of the electric field at the horn opening was perpendicular to the ground, and the two horns moved horizontally along the ground in the bistatic RCS measurement. *θ* is the incident angle under TE polarization, and d is the distance from the diffusion metasurface to the transmitting and receiving horn antennas, which met the far-field test conditions. The sample was placed in the middle spot between the two horns. The horns needed to be moved to ensure that the TE incident angle and TE reflection angle of the two horns to the sample were the same. In the reflection coefficient measurement, a foam board was used to support the sample shown in Figure 6c. To approach the ultrawideband performance of the metasurface, four sets of linearly polarized horn antennas working within 8.2–12.4, 12.4–18, 18–26.5, and 26.5–40 GHz were utilized as the receiver and transmitter [26].

To eliminate the environment interference, the experiments adopted the time-domain gating function of the network analyzer as a method. The polarization of the transmitting antenna and the receiving antenna were always the same, and the distance between the sample and the antenna met the conditions of the far-field test. For both bistatic and monostatic RCS measurement, a same size metal plate should be placed to evaluate the RCS reduction of the metasurface.

Figure 6d shows the simulated and measured monostatic RCS reduction curves of the proposed diffusion metasurface at normal incidence under TE and TM polarizations. From the results, the bandwidth of monostatic RCS reduction under TE and TM polarizations (greater than 10 dB) was 7.1–29.2 GHz, which is in good agreement with the simulated results. The slight difference between simulated and measured RCS reduction results at higher frequencies was likely caused by the errors in manufacturing and assembling the prototype.

The mirror bistatic RCS reduction under the TE polarization with incident angles of *θ* = 15°, 30°, and 45° is shown in Figure 6e. A more than 10 dB RCS reduction from 7.1 to 29.2 GHz was observed. Numerical values and experimental results were consistent with each other. The simulation frequency sweep step was set to 1 GHz because of the large matrix arrangement. During the measurement, the frequency sweep step of the vector network analyzer was 0.1 GHz. Since there were more frequency points, the measured result looked smoother compared to the ones for the numerical. As for the amplitude difference of simulation and measurement, it was caused by a suboptimal experimental environment under large oblique incidence.

Table 1 lists the comparison between the proposed diffusion metasurface and other similar designs. A figure of merit (FoM) [27] is provided to describe the relationship between bandwidth performance and thickness, where FoM = BW(BWR)/T, BW = 2 × (fH − fL)/(fH + fL), BWR= fH/fL, and T is the electrical thickness at the lowest operating frequency. From Table 1, it can be concluded that the proposed diffusion metasurface had an ultrawide bandwidth radio and the largest FoM in polarization-insensitive designs.

## 4. Conclusions

In summary, a polarization-insensitive diffusion metasurface with ultrawideband backscattering reduction property was proposed in this paper. The metasurface was constructed by coding two types of 180° phase-difference elements, and the “0” element was constructed by period-changed unit cells to obtain the ultrawideband characteristic. An annealing algorithm was employed to seek an optimal sequence of the metasurface unit cells. To verify the new method, an ultrawideband diffuse metasurface was simulated, fabricated, and measured. Both the simulated and measured results demonstrate that the metasurface efficiently decreased the monostatic backscatter RCS (by more than 10 dB) within 7.1–29.2 GHz compared to the equal-sized PEC plane. The benefit of this design method is that it is more effective than other diffuse metasurface designs. On the simulated metasurface, the bandwidth of RCS reduction increased by 122% compared to other classical arrangements and diffusion metasurface designs. Furthermore, the scattering energy was reflected in many directions in the ultrawideband.

## Figures and Tables

**Table 1:** Comparison With Other Wideband RCS Reduction Design.

Ref.	Freq. (GHz)	BW/ BWR	Polarization -Insensitive	Thick (λ_0_)	FoM
[16]	6.4–23.5	114/3.67	no	0.07	16.32/52.43
[17]	7.9–20.8	89.9/2.63	no	0.079	11.38/33.29
[23]	8.4–22.7	92.2/2.71	yes	0.084	10.98/32.14
[24]	3.75–10	91/2.67	yes	0.079	11.52/33.8
[26]	6.1–17.8	98/2.92	no	0.12	8.16/24.3
[28]	7.79–30.22	118/3.87	no	0.091	12.96/42.52
[29]	16.5–58	111.5/3.52	yes	0.165	6.76/21.33
This work	7.1–29.2	122/4.14	yes	0.105	11.62/39.43

## Data Availability

Data sharing is not applicable.
